# Sheehan’s Syndrome Presenting as Major Depressive Disorder

**Published:** 2015-01

**Authors:** Mehmood I Qadri, Mohsin Bin Mushtaq, Iram Qazi, Sameena Yousuf, Aaliya Rashid

**Affiliations:** 1 Department of General Medicine, Sher-e-Kashmir Institute of Medical Sciences, Medical College Hospital, Srinagar, India;; 2 Department of Ophthalmology, Shri Maharaja Hari Singh Hospital, Srinagar, India;; 3 Department of Preventive and Social Medicine, Sher-e-Kashmir Institute of Medical Sciences, Soura, Srinagar, India;; 4 Division of Phaco Surgery, Al-Kabir Medical Center, Srinagar, India

**Keywords:** Hypopituitarism, Postpartum hemorrhage, Major depressive disorder

## Abstract

Sheehan’s syndrome or Simmond’s disease is a rare endocrine disorder seen in clinical practice. The clinical spectrum is diverse and a high index of suspicion together with a good clinical acumen and proper diagnostic approach helps in early diagnosis and prompt treatment of this endocrinopathy. Sheehan’s syndrome presenting as a major depressive disorder finds less mention in the literature.

The patient discussed here is a 45-year-old female who had been on antidepressants and psychiatry follow up for a long time until she presented to our Out Patient Department (OPD), where she was evaluated in detail and diagnosed as a case of Sheehan’s syndrome. The patient is doing well and is on a regular follow-up with us. Further studies are required to demystify the strength of this association in more detail and to elucidate the possible underlying mechanism.

## Introduction

Sheehan’s syndrome is the cause of 0.5% of all cases of hypopituitarism in women. It is caused by necrosis of the pituitary gland usually following severe hypotension or shock caused by massive hemorrhage during or after delivery. Other possible etiologies include vasospasm, thrombosis, and compression of hypophyseal arteries. Physiological enlargement of the pituitary gland during pregnancy, a small sella, and disseminated intravascular coagulation has been suggested to play some role in its etiopathogenesis.

Patients with Sheehan’s syndrome have varying degrees of anterior pituitary hormone deficiency.^1^ The usual sequence of hormone depletion is Growth Hormone (GH), Follicle Stimulating Hormone (FSH), Luteinizing Hormone (LH), Thyroid Stimulating Hormone (TSH), and Adrenocorticotropic hormone (ACTH).

Sheehan’s syndrome is one of the common causes of hypopituitarism among women in developing countries, although its incidence in developed countries is declining owing to well-advanced obstetric care. A prevalence rate of 3% has been reported in Kashmir valley in women aged 20 years and above.^2^ A study conducted in Iceland showed a prevalence rate of 5.1 per 100,000 women.^3^ Sheehan’s syndrome has a gradual onset and a slow, indolent course. This is a major reason why most of cases are diagnosed late and some labeled with alternative diagnoses. Therefore, in suspected cases, a detailed history regarding postpartum bleeding, lactation failure, and cessation of menstrual cycles is indispensable to arrive at an early diagnosis of Sheehan’s syndrome and thereby reduce the morbidity and mortality associated with this disorder. We present this case report to point out the various fallacies in the management of such patients, which can lead to inappropriate treatment regimens, delay, and worsening of the symptoms. Written informed consent was obtained from the patient for publication of this case report.

## Case Presentation

A 45-year-old female was admitted in our hospital with a one-year history of generalized weakness, easy fatigability, loss of appetite, generalized body aches and pains, malaise and chronic ill health. 

She had consulted several practitioners and been diagnosed as a case of major depressive disorder and hypothyroidism. She had been prescribed a number of antidepressants and Thyroxine. Detailed history revealed that the onset of symptoms had been 8 years earlier when she had her last child. It was a full term vaginal delivery at home. The patient denied any history of profuse bleeding postpartum. However, she had lactation failure and cessation of her menstrual cycles after the delivery. 

Physical examination at the time of admission revealed an ill-looking patient with cold extremities and torso, feeble pulse with a rate of 54 bpm, blood pressure 80/60 mmHg in lying down and 68/ 50 mmHg in sitting posture; with postural drop (+), respiratory rate of 16/min, temperature (oral) 36.9ºF. The patient had pallor, coarse dry skin, non-pitting edema of lower limbs, sparse eyebrows and eyelashes, atrophied breasts, very scanty body hair and a hoarse voice. Chest and cardiovascular examinations were normal. Abdominal examination revealed diffuse tenderness. The patient was conscious, mentation, however, was slow. CNS examination revealed sluggish Deep Tendon Reflexes and a typical hung-up reflex was observed in the ankles. 

Laboratory parameters showed anemia (normocytic, normochromic), blood sugar (random) 40 mg/dl, urea 26 mg/dl, serum creatinine 1.0 mg/dl, serum sodium 144 mEq /L, serum potassium 3.6 mEq/L, serum calcium 8.0 mg/dl. Liver function tests, chest roentgenogram, and USG abdomen were normal. ECG showed low voltage QRS complexes, sinus bradycardia, and prolonged QT interval (QTc=0.48 seconds). Routine urine examination revealed 8 to 10 pus cells without any evidence for the presence of sugar or albumin. The hormone profile showed serum cortisol (8.00 AM) 3.17 ug/dL (normal range 4.30-22.40), serum TSH 3.12 mIU/ml (normal range 0.5-6.5), serum FSH 3.00 mIU/l (normal range 2.0-6.6), serum LH 0.42 mIU/l (normal range 3.0-12.0), serum PRL 0.86 ng/ml (normal range >2.0) and serum GH 0.22 ng/ml (normal range >3.0). An impression of hypopituitarism was made based on history, physical examination and laboratory parameters (biochemical and hormonal).

The patient was managed with I.V. fluids (dextrose/normal saline) and hormone replacement therapy in the form of injection Hydrocortisone (50 mg I.V. 6 hourly) and Thyroxine at a dose of 50 mcg/day. The patient was also put on antibiotics (for UTI) and calcium supplements. Her general condition showed a dramatic improvement; appetite improved, blood pressure showed an upward trend and body and extremities became warm. Blood glucose levels increased from baseline 40 mg/dl to 165 mg/dl (random). The patient was switched to orals, and hydrocortisone was changed to tablets. Prednisolone 5 mg (morning), and 2.5 mg (evening), while Thyroxine continued at the same dose. The patient was discharged on the 7th day of admission and advised to continue the same medication at home.

**Figure 1:**
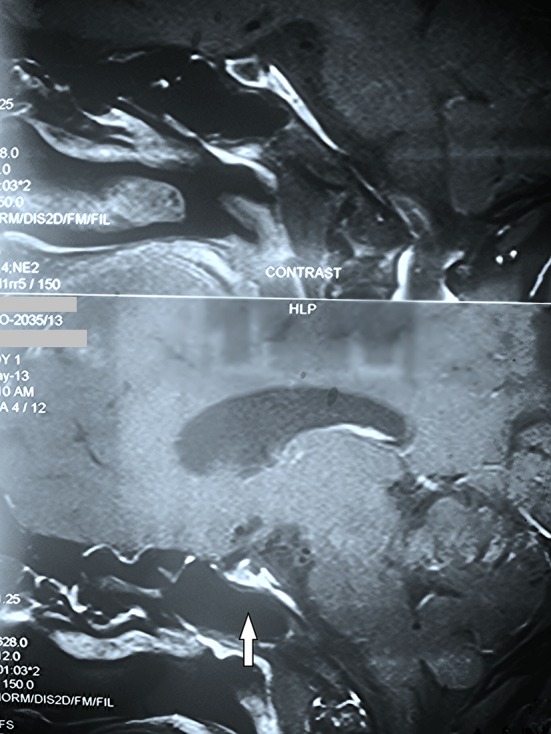
Sagittal view MRI of the patient revealing empty sella (arrow).

MRI brain of the patient was done on follow up, which revealed an* empty sella* (figure 1). The rest of the scan was normal.

## Discussion

Sheehan’s syndrome, first described by Sheehan in 1937,^4^ is a postpartum hypopituitarism caused by necrosis of the pituitary gland. It is usually the result of hypotension or shock caused by massive hemorrhage during or after delivery. It can present in the postpartum period as lactation failure or may manifest several months or years following delivery. A study has mentioned an average span of 13 years between the obstetric event and diagnosis.^5^ The presentation can be varied and include lactation failure following delivery, cessation of menstrual periods, generalized weakness and debility, premature wrinkling of the forehead and face, genitals and body hair loss, and coarse dry skin. Rare clinical presentations include acute circulatory collapse, congestive cardiac failure, hypoglycemia, diabetes insipidus, or psychosis.^6^^-^^9^ The usual sequence of anterior pituitary hormone depletion is GH, FSH, LH, TSH and ACTH. The main involvement is in the secretion of GH and Prolactin (approximately 90% to 100%).^5^^,^^10^ A study has revealed a co-relation between growth hormone deficiency and psychiatric manifestations with the most frequent psychiatric diagnosis being major depression (32% of GHD patients) and dysthymia.^11^

The clinical manifestations become evident when at least 75% of pituitary is damaged. The lactation failure is common and the lack of the prolactin response to administration of TRH has been suggested as a sensitive test for screening of patients with suspicion of Sheehan’s syndrome.^12^ The most common electrolyte abnormality encountered is hyponatremia, seen in 33% to 69% of all cases.^13^ Clinical diabetes insipidus is an uncommon complication^6^ although posterior pituitary functions have been shown to be impaired in Sheehan’s syndrome.

Antipituitary antibodies have been demonstrated in some patients.^14^ However, their exact role in etiopathogenesis is not clear.

Neuroimaging of the pituitary shows an empty sella in about 70% of patients, or partially empty sella in about 30% of patients. These findings on MRI characterize Sheehan’s syndrome and provide early confirmation of the clinical diagnosis.^15^^,^^16^

Treatment consists of lifelong hormone replacement. Glucocorticoids should be replaced before the replacement of thyroid hormone. Patients with diabetes insipidus are put on Desmopressin (DDAVP).^12^

As the clinical features of Sheehan’s syndrome are often subtle, years may pass before a definitive diagnosis is made.^1^ Our patient had been diagnosed as a case of major depressive disorder and put on antidepressants and Levothyroxine. Thus, she was partially treated leading to a delay in diagnosis and worsening of her symptoms. Therefore, a detailed history of postpartum hemorrhage, failure to breastfeed the baby and cessation of menstrual periods are vital clues to an early diagnosis, which would be instrumental in the initiation of appropriate treatment in order to reduce the morbidity and mortality associated with this disorder.

## Conclusion

In conclusion, apart from the already-known associations of Sheehan’s syndrome, clinicians must always keep in mind that a combination of Sheehan’s syndrome with major depressive disorder could exist. In view of this rare case, emphasizing the magnitude of the problem, clinicians should appropriately evaluate patients with Sheehan’s syndrome and its associated cluster of abnormalities as a part of standard care for these patients. Nevertheless, more research needs to be done to unravel the cryptic etiopathogenetic causes for this rare association and new effective therapeutic strategies be evaluated to prevent patients from untoward consequences of such disastrous -if not managed in time- health issues. 
